# Panel Servitude without Anæsthetics

**Published:** 1913-03-29

**Authors:** 


### The Hospital

The Workers' Newspaper of Administrative Medicine and Institutional Life, Administration, National Insurance and Health.

No. 1395, Vol. LIII. SATURDAY, MARCH 29, 1913. PRICE ONE PENNY.

### PANEL SERVITUDE WITHOUT ANÆSTHETICS.

As soon as ever the regulations of the Insurance Commissioners dealing with medical benefit under the panel system were published we remarked in these columns on certain of the arrangements which were bound to work badly and to result in grievous hardship to the insured. That this has been the case since January 15 the daily papers have borne frequent witness; and a good many of the inquests at which such facts have emerged have been briefly reported in The Hospital. From the very outset we emphasised most strongly the folly and injustice of making the panel practitioner who undertakes any operation on an insured patient himself liable for the fee of the anæsthetist. This absurd provision has not yet, so far as we know, rendered necessary an inquest; but that it is producing the dire results we foretold is obvious from the following narrative, brought under our notice in so direct a manner that we can guarantee the main facts to be accurate.

An insured person, a girl of some twenty years of age, or thereabouts, developed during the second week of March a very painful swelling of the breast; in fact, an acute abscess. She went to her panel doctor, accompanied by an older woman. The doctor diagnosed the case correctly (it was impossible not to do so); and prepared to treat it by incision, which was also correct. Without proper antiseptic precautions, according to the patient's friend, who was an eye-witness, he plunged a knife into the swollen breast; no anæsthetic, local or general, was employed. The incision failed to reach the abscess cavity, and no pus came out; but the doctor applied a dressing and dismissed the patient. After a night of suffering, another doctor, not on the panel, was consulted, who opened the abscess under full anæsthesia with all due observance of Listerian principles.

So much for the facts of the case (not the only one of its kind we could quote), eloquent enough in all conscience as they are. What of their application? To begin with, we shall dismiss from consideration the alleged neglect of antiseptic treatment, criminal though it was if the eye-witness' statement is correct. About this there is the possibility of mistake; and in any case it reflects upon the individual paneller, not upon the panel system. But the omission to anæsthetise the patient is quite on another footing. How any doctor could bring himself to stab in this way a woman affected with that acutely painful disease, breast abscess, we cannot understand. Such a proceeding is rank bad surgery in every way for one thing; and gross inhumanity for another. We do not in the least excuse his cruelty; but we do say that the panel system is responsible for it. That system penalises the honest and capable among the panel doctors, and subsidises those who are neither. Those who shirk all operative work, even such simple procedures as the incision of abscesses, get their pay all the same without having fairly earned it; whereas those who act up to a reasonable standard of efficiency not only have to do more work, but are actually directly out of pocket! Could anything be devised more surely to premiate incompetence and cruelty?

We can readily imagine what form the explanations of the National Insurance Commissioners will take. They will say that this particular patient has only to prefer a complaint about her treatment, and that it will be fully investigated. But to begin with, she will most probably not complain; for this means further bother about a matter which she would prefer to forget as speedily as possible. Moreover, the circumstances we have narrated are much less important as evidence that a panel doctor has behaved like a ruffian, than as *proof that the panel system as now administered is disastrous.* So long as panel practitioners are out of pocket through performing operations under anæsthesia, so long will they think twice about giving their patients the benefit of it. We trust and believe that there are few who in a similar case would behave as this particular doctor did; it is quite impossible to deal properly with an acute breast abscess without full general anæsthesia, apart from the question of its inhumanity. But there are borderline cases in which it is legitimate to do slight operations under local anæsthesia, or even none at all, when the patient is stoical. And the tendency will be to treat all patients, including the most delicate and nervous, and including children, as if they were as little sensible to pain as the average "shellback." In this way a great deal of real and avoidable suffering will be created, traceable directly to the regulations about medical benefit.

No reasonable person will deny that the insured have good reason to demand the repeal of this monstrous regulation. Their blood and tears have been weighed in the balance against a trifling saving of the money wrung from them by an Act they do not approve of; and economy has won the day. We present this reflection and example to Mr. Lloyd George and to the Insurance Commissioners. The latter, at least, can hardly disclaim their responsibility; for this very point about anæsthetics was put before them by the General Medical Council on December 13, and the Commissioners refused to make any alteration in their regulations. The case we have cited above is not the only one of the sort of which we have knowledge; and we want to know how long this sort of thing is to go on. It is for the Insurance Commissioners to answer.

